# Nanoscopic origin of cracks in carbon fibre-reinforced plastic composites

**DOI:** 10.1038/s41598-019-55904-2

**Published:** 2019-12-17

**Authors:** Masao Kimura, Toshiki Watanabe, Yasuo Takeichi, Yasuihiro Niwa

**Affiliations:** 1 Photon Factory, Institute of Materials Structure Science, High Energy Accelerator Research Organization (KEK), Tsukuba Ibaraki, 305-0801 Japan; 2 Department of Materials Structure Science, School of High Energy Accelerator Science, SOKENDAI (Graduate University for Advanced Studies), Tsukuba Ibaraki, 305-0801 Japan

**Keywords:** Composites, Microscopy

## Abstract

Voids and cracks can fatally degrade structural materials such as metals and ceramics but are tolerated in carbon fibre-reinforced plastic (CFRP) composites if monitored to prevent their growth to a critical size. Thus, the use of CFRPs as aeronautical structural materials requires an understanding of microscopic crack formation. However, this crack-formation mechanism remains unclear because experimental difficulties have hindered studies of relevant phenomena that occur *before* crack formation. Herein, we report high-resolution (~50 nm) and non-destructive three-dimensional observations of crack initiation and propagation under applied stress. This evaluation reveals that voids and cracks do not simply result from local stresses but instead occur largely through two competing nanoscale mechanisms, namely, *fibre/plastic interface debonding* and *in-plastic crack initiation*. Therefore, nanoscopic insights into these heterogeneities are essential for controlling crack initiation and determining reasonable safety margins for CFRP composite use.

## Introduction

Structural materials such as steel, ceramics, and composites are commonly subjected to multiple loads that can cause serious damage. Their failure is always associated with the presence of defects on various scales, and the initiation and propagation mechanisms of these defects vary broadly according to differences in material bonding^1–3^. For example, the mechanical properties of steel and metals largely depend on the movement of dislocations (*i.e*. topological line defects) or successive atomic displacements^1^.

**Figure 1:**
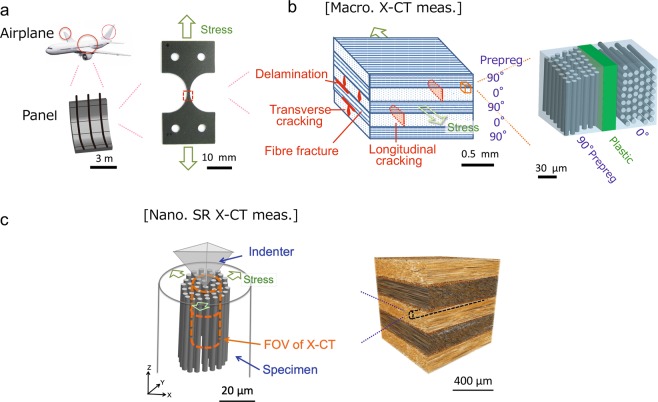
Multiscale structures of CFRPs and their observation with multiscale X-CT. (**a**), CFRPs used as aeronautical structural materials for the wings and body of an airplane. An example CFRP with a layup of [90°/0°/90°/0°/90°], as used in this study. (**b**), Schematic representation of a cross-section of CFRP (left), and a part of magnified image. When mechanically loaded, different failure mechanisms are induced at a macroscopic scale (see the text). (**c**), (right) X-CT image of the part of the CFRP used in this study. A columnar specimen (broken black lines) was mechanically cut from a single ply layer for the nanoscopic SR X-CT measurements. (left) Schematic illustration of the nanomechanical test stage for *in situ* SR X-CT measurements (Methods). The FOV in the nanoscopic SR X-CT measurements (orange broken lines) was much smaller than the specimen dimensions (grey lines), which eliminated surface effects in the acquired image. *The aeroplane image in* Fig. 1a *was obtained from the pamphlet of the national project: the Structural Materials for Innovation of the Cross Ministerial Strategic Innovation Promotion Program (unit D66 in SM*^4^*I, SIP) of the Japan Science and Technology Agency (JST) under the permission by JST*.

By contrast, carbon fibre-reinforced plastic (CFRP) composites have multiple components, that is, strong but brittle anisotropic carbon fibres together with a ductile and isotropic plastic, held together by adhesive bonding. During CFRP production, the carbon fibres are usually aligned in the same direction and pre-impregnated with the plastic to form ‘prepreg’ sheets, which are then sequentially stacked at various relative orientations, *e.g*. 0°/90°/0°… or 0°/45°/90°… (Fig. 1a,b). These lightweight composites are extensively used to reduce the weight of structural materials in the aerospace industry, such as in the wings and bodies of airplanes or in automobiles^4,5^. The mechanisms of crack initiation and propagation in CFRP composites have been investigated using various modelling and analysis techniques. Classical macroscopic models assume that all bodies contain cracks and voids, and that the growth of these defects is stable when the released strain energy exceeds the energies required to create a new surface area and generate plastic deformation near the crack tip^6,7^. When CFRPs are subjected to mechanical loading, different failure mechanisms are induced at the macroscopic scale^8–11^: (M1) transverse matrix cracking in 90° plies, (M2) longitudinal matrix cracking in 0° plies, (M3) delamination between 0° and 90° plies, and (M4) fibre fractures in 0° plies (Fig. 1b). In the case of uniaxial loading, the early state of damage is dominated by the mechanism M1^12^. However, most previous investigations focused on heavily damaged stages in which crack propagation was sufficient for observation (larger than a few to a few hundreds of micrometres).

Experimentally, cracks in CFRPs have been investigated using various techniques^13^. For example, infrared thermography and radiography can inspect large areas in short times. Ultrasonic inspection detects the presence of flaws, though the surface has to be accessible to probe^13^. Acoustic methods and optical fibre testing are used for continuous monitoring^13^. Whereas these methods detect only large cracks (> about few mm), X-ray computed tomography (X-CT) methods have provided substantial insight into the mechanics of damage in three dimensions, including the macroscopic mechanism of crack initiation and propagation^14^. In X-CT methods, a series of digital radiograph projections are measured with a sample rotated by a small increment between each image, and a three-dimensional (3D) image of the contrast variation was reconstructed mathematically^14^, with merits of being non-destructive and relatively high spatial resolution (*ca*. submillimetre). Recently, a non-destructive X-CT method using synchrotron radiation was developed that can observe cracks inside the bulk with a spatial resolution as small as a few micrometres^15,16^.

However, owing to recent advances in the production of CFRPs through microscopic design, observation with even higher spatial resolution of less than a micrometre is required in order to understand the mechanism of crack initiation and propagation. It has been reported that the propagation of delamination caused by impact can be reduced by non-uniform resin layers^17^, dispersion of secondary phase particles^18^, or electrospun thermoplastic nanofibers^19^ within the interlaminar regions, where inhomogeneity in the plastic (resin) exists on a scale of 1–10^2^ μm. An investigation of the crack path in a particle-toughened interlayer within a polymer composite laminate showed that Mode I crack propagation involves a discontinuous process zone^16^. Higher interlaminar fracture toughness, both under Mode I and Mode II loading conditions, has been attributed to the bridging of (micro)cracks by polyamide nanofibers^20^. Modification of the carbon fibre surface has been attempted using various techniques to control the microscopic structure, such as sizing, plasma, or chemical treatments and carbon nanotube or nanoparticle coatings, to increase the wettability and interfacial adhesion with polymeric matrices^21^.

Various theoretical approaches have been used in attempts to analyse these microscopic processes and their failure mechanisms. Finite element modelling and analysis (FEM) have been exploited to understanding the details of failure mechanisms such as mechanisms M1–M4 described above^22,23^. An investigation of interlaminar damage propagation using a cohesive zone model with automatic mesh generation showed that the damage was formed at the centre of the lowermost ply with butterfly-shaped delamination^24^. The effects of fibre array irregularities on microscopic interfacial stress states have been investigated using observed image-based models, which showed that the stress reaches a maximum at a certain interfibre distance and suggests possible locations of crack initiation^25,26^. Statistical strength theory has been developed to understand the variability in strength by modelling the local stress fields around the many interacting fibre breaks^27^. The fracture properties, such as strength and toughness of the fibre–matrix interface, have been investigated using the fragmentation process and debonding growth in single-fibre composites^28,29^. Furthermore, the stress–strain curves of polymer–carbon nanotube composites have been calculated using a molecular dynamics simulation for comparison with the corresponding rule-of-mixture predictions^30,31^. These theoretical approaches have suggested that the inhomogeneities in mechanical properties, strains, cohesive energies, etc. at the microscopic scale (sub-micrometre) are key for crack initiation and propagation. Therefore, non-destructive and 3D observation techniques with better spatial resolution (sub-micrometre) are required for the development of both theoretical micromechanical approaches and advanced processes for CFRP production. Observation of cracks with such spatial resolution was achieved using scanning electron microscopy (SEM)^8,10,25,32^ or SEM combined with a focused ion beam (FIB)^32^. However, these techniques either detect only the surfaces of specimens or are destructive. Therefore, crack initiation and propagation has never been directly observed at a scale of less than a micrometre because of experimental difficulties.

Here, we non-destructively observed crack initiation and propagation on multiple scales. First, we examined cracking by *in situ* observations with the macroscopic X-CT. However, the relatively small density difference between carbon fibres and the plastic matrix in CFRPs makes it challenging to obtain high-contrast images at a smaller scale (sub-micrometre) using conventional absorption-contrast X-CT measurements. Thus, we utilized a technique developed for observing crack initiation with a spatial resolution of ~50 nm, namely, nanoscopic X-CT using synchrotron radiation (SR X-CT), which couples phase-contrast X-CT and transmission X-ray microscopy using synchrotron radiation. The high resolution in this study was achieved using a full-field imaging technique involving advanced X-ray optical components and phase contrast imaging^33–35^ (Methods).

## Results

Considering the hierarchical structure of CFRPs, the first step of crack initiation under a tensile stress was identified. The obtained results showed that transverse cracks (a few hundred micrometres) first appeared in the 90° plies at ~40–50% of the fracture strength (*σ*_*f*_), and their propagation accelerated several times with an increase in tensile stress (Supplementary Note 1). Thus, we focused on crack initiation in the 90° plies and investigated the details of crack initiation at the nanoscale. A columnar specimen with unidirectional carbon fibres was mechanically loaded with a diamond indenter using a nanomechanical test stage^36^ (Fig. 1c), and *in situ* observations under stress were performed by nanoscopic SR X-CT.

**Figure 2:**
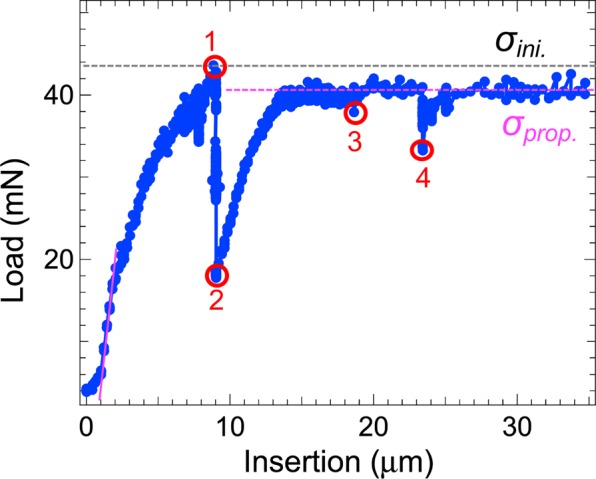
Typical nanomechanical specimen testing data corresponding to the strain–stress curve. In the region of low tensile stress, the indentation force first increases near linearly (pink line) and then according to a power law with increasing insertion distance. The load drops sharply at point 1 and at points 2,3,4 when it reaches the critical values: *σ*_*ini*._ and *σ*_*prop*._, respectively. The drops at points 1 and 2,3,4 may correspond to initiation and propagation of cracks within the specimen, respectively.

**Figure 3:**
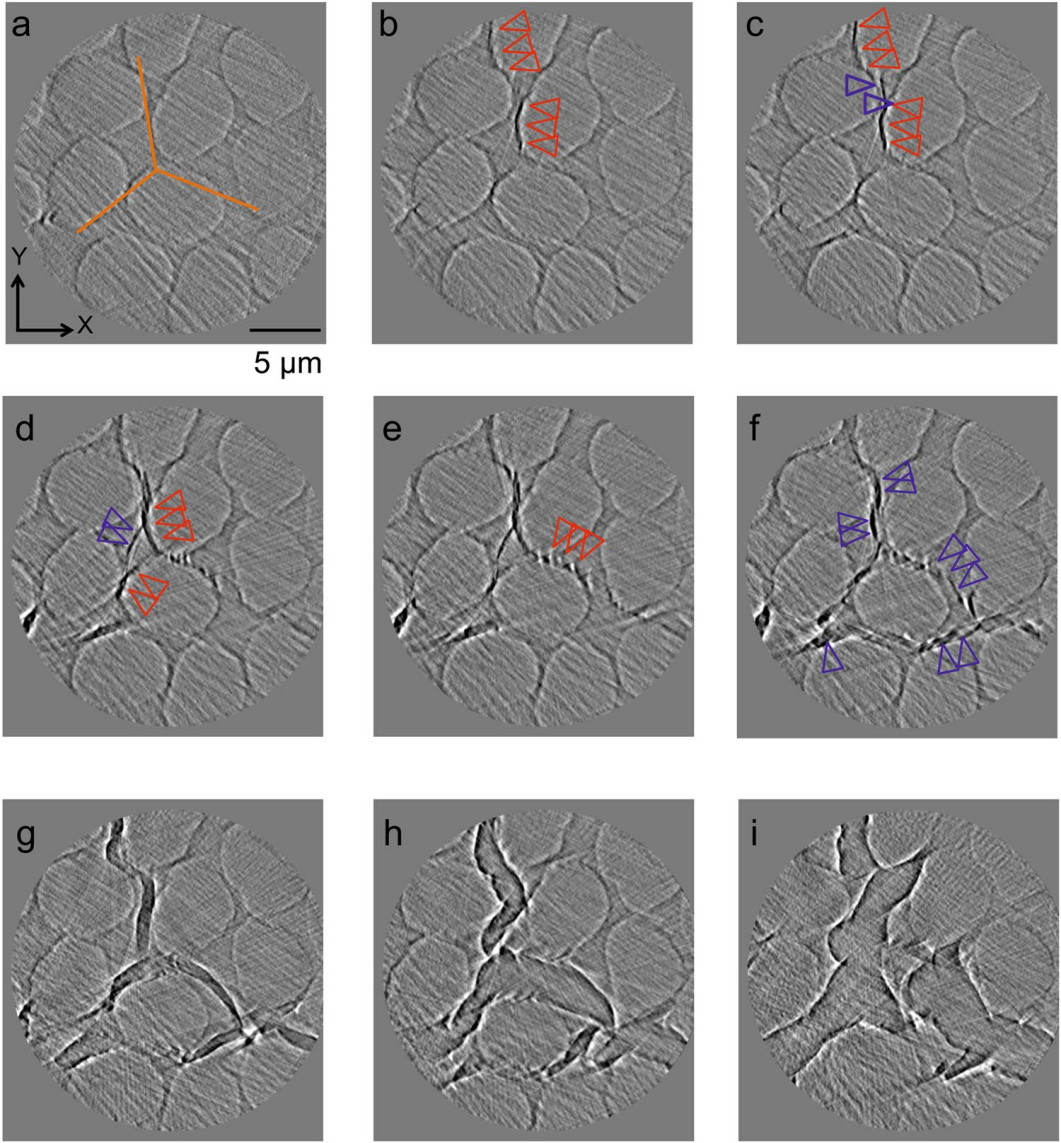
Cross-sectional images in the *X*-*Y* planes showing crack propagation. The stress was applied by a pyramidal diamond indenter to the initial position of the pyramidal indenter wedge (orange lines in **a**). The scale bar in (**a**) is 5 μm. (**a–i**), cross-sectional images at various positions along the *Z*-axis of the specimen ((**a**), 0.0; (**b**), 2.0; (**c**), 4.0; (**d**), 5.0; (**e**), 8.0; (**f**), 10.0; (**g**), 18.0; (**h**), 20.0; and (**i**), 30.0 μm) corresponding to different strains; the nominal strain was ~13% at *Z = *20 μm and ~0% at 0 μm. Triangles mark regions where cracks are initiated and propagate along fibre/plastic interfaces (red) and in the plastic matrix (blue).

**Figure 4:**
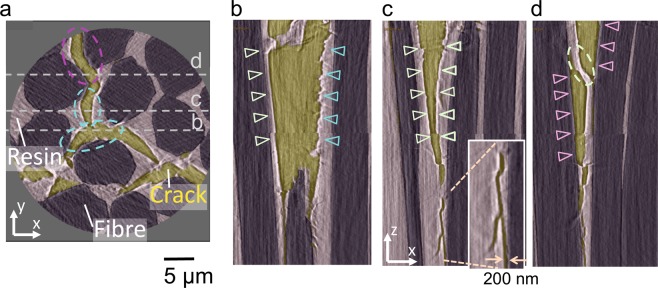
Segmented images of cracks formed in the CFRP. (**a**), *X*-*Y* and (**b**, **c**, **d**), *X*-*Z* cross-sectional images corresponding to sections along broken lines b, c, and d in (**a**). Carbon fibres, plastic resin, and cracks (air) are shown in purple, grey, and yellow, respectively. (**b**,**c**) typical *in-plastic crack initiation* with smooth (green triangles) and rough (blue triangles) crack surfaces. (**d**) *fibre/plastic interface debonding* (red triangles). Branching at the crack tip (inset in **(c**)) and plastic bridges between interfaces (broken line in (**d**)) are also clearly observed.

**Figure 5:**
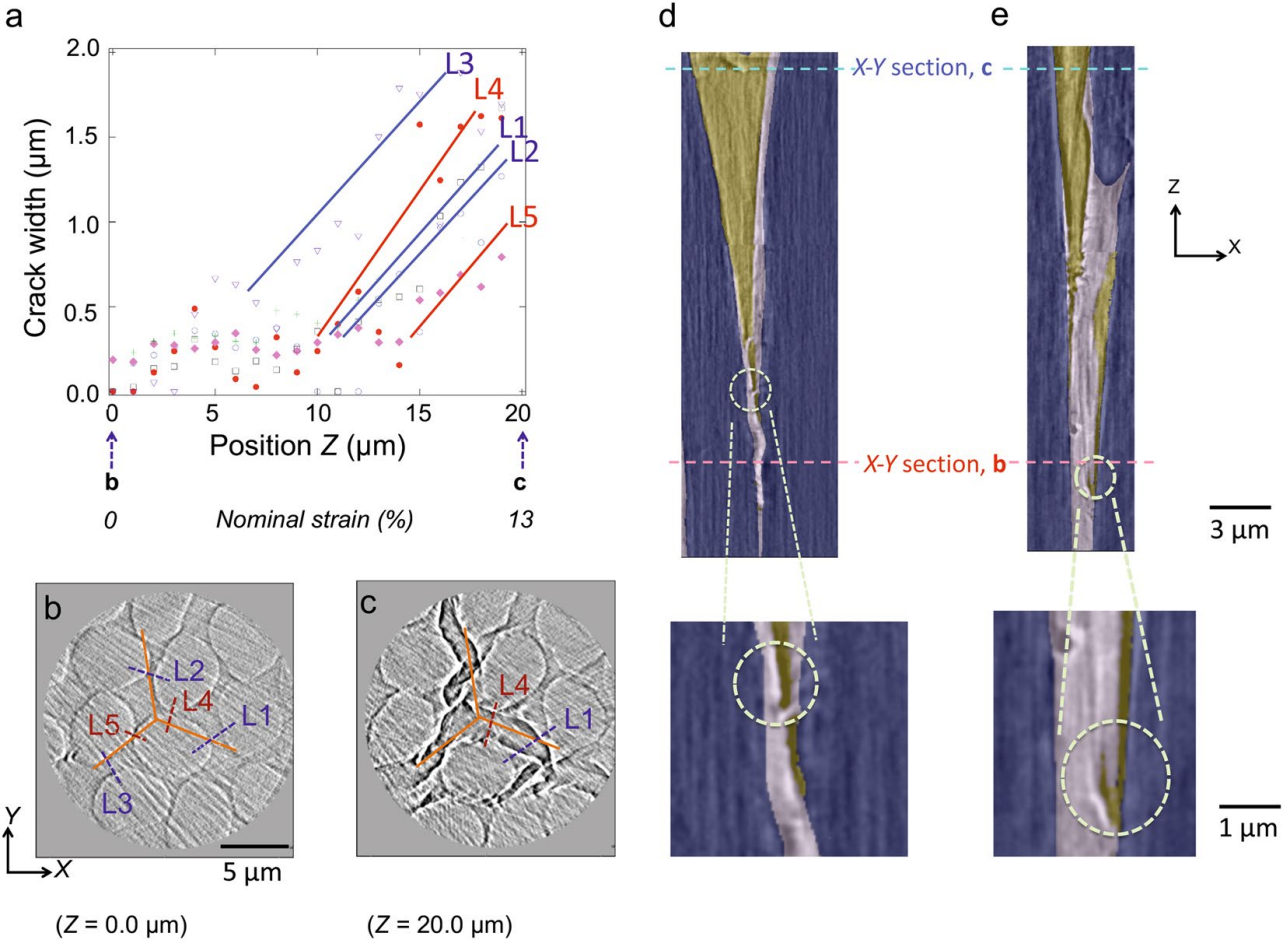
Crack propagation susceptibility at different locations. (**a**), Crack width along the *Z*-axis (propagation direction, see Fig. 1c) at different locations, denoted L1–L5 in (**b**). (**b**,**c**), *X*-*Y* cross-sectional images at *Z* = 0 (zero strain) and 20 μm (the maximum strain), respectively. The orange lines show the initial position of the pyramidal indenter wedge. (**d**,**e**), Cross-sectional images at locations L1 and L4 in (**b**,**c**), showing a crack that propagated by *in-plastic crack initiation* (**d**) and *fibre/plastic interface debonding* (**e**). Carbon fibres, plastic resin, and cracks (air) are shown in purple, grey, and yellow, respectively.

First, it was confirmed that nanomechanical testing successfully caused the initiation and propagation of a crack within a specimen. Figure 2 shows a typical example of the measured load versus the insertion distance, corresponding to a strain–stress curve, although the measured load is not exactly equal to the strength in the case of indentation. In the region of low tensile stress, the indentation force first increased nearly linearly and then according to a power law with increasing insertion distance. These increases may correspond to consecutive elastic and plastic deformation processes, respectively. When the insertion distance was increased further, the force reached a maximum value (the load for the first initiation: *σ*_*ini*._, ~44 mN, ‘1’ in Fig. 2), then decreased sharply. At this point, a new crack was initiated accompanying a release of the load (or strain), as confirmed by two-dimensional (2D) time-lapse observations (Supplementary Note 2). When the load stopped decreasing (‘2’ in Fig. 2), the initiated crack was considered to reach a stable state. Then, nanoscopic SR X-CT measurements were performed with the specimen rotated, which took approximately 1 h. The details of the measured images are shown in Figs. 3–5.

After each nanoscopic SR X-CT measurement, the insertion distance was increased gradually (~20 μm/h). The load reached another maximum (the load for the propagation: *σ*_*prop*._, ~41 mN) and then underwent sudden drops (‘3’ and ‘4’ in Fig. 2) as the insertion distance increased. The 2D time-lapse observations confirmed that the crack propagated stepwise at these drops. The *σ*_*prop*._ value corresponds to the local critical stress that is required to propagate an initiated crack within a specimen under a constantly increasing applied stress. The *σ*_*prop*._ value is approximately 93% of the *σ*_*ini*._ value, likely because the stress required to propagate an existing crack is smaller than that to initiate a crack. Furthermore, the amount by which the load dropped during the second drop was small compared with that during the first drop. This difference may be because the mechanical load was applied by indentation, which created a complicated stress field, especially at high strain, and the friction between the indenter and the specimen may differ depending on the insertion distance.

These observations clearly showed that the initiated crack propagated stepwise with steps of a few micrometres when the force reached a critical value of *σ*_*ini*._ or *σ*_*prop*._. This phenomenon was observed repeatedly for more than ten different specimens. This stepwise propagation, which was directly observed here for the first time, is an intrinsic feature of CFRP mechanical failure. It differs considerably from phenomena witnessed in metals^1^, where even small cracks rapidly propagate through a specimen and consistently cause fatal macroscopic failure. Therefore, the above stepwise propagation should reflect nanoscopic processes related to accumulated strain energy release and temporary prevention of cracking progress, as described below.

Subsequently, we paused the nanomechanical testing to increase the insertion distance of the indenter tip and performed nanoscopic X-CT measurements to observe the 3D details of crack initiation and propagation. Figure 3 shows a series of cross-sectional images of volume data for *X*-*Y* planes along the *Z*-direction (*i.e*. corresponding to various strains) when the cracks reached a stable state (‘2’ in Fig. 2). The obtained images were clear enough to be segmented (Fig. 4 and Supplementary Movie S1). To the best of our knowledge, this is the first example of *in situ* and non-destructive observations of crack initiation and propagation within a CFRP at a high resolution of ~50 nm. It should be stressed that the 3D images obtained here show the crack behaviour inside the specimen, which is free of surface effects. Such observations are not possible with conventional techniques such as SEM and previous X-CT methods.

At the nanoscale, the initiation of cracks was location-dependent and heterogeneous, and two competing processes, defined as *fibre/plastic interface debonding* and *in-plastic crack initiation*, were observed. In the *fibre/plastic interface debonding* process, cracks were initiated by opening the fibre/plastic interfaces measured ~100–200 nm and propagated along the fibre/plastic boundary (red triangles, Fig. 3b–e). This debonding process may depend on the fibre/plastic bonding energy and, as a result, can be affected by fibre surface treatments and the surface morphology. In later deformation stages, cracks propagated along the fibre/plastic interface in the *Z*-direction and spread between neighbouring fibre/plastic interfaces. These surfaces exhibited sharp crack boundaries (red triangles, Fig. 4d) and plastic bridging occurred (broken line, Fig. 4d).

On the other hand, *in-plastic crack initiation* was also observed, where small voids (blue triangles, Fig. 3c,d,f) were initiated and propagated via plastic deformation. These cracks travelled across the plastic matrix to neighbouring fibres, propagated within the plastic in the *Z*-direction along a path with rough or smooth crack surfaces, or branched into two or more cracks (Fig. 4b,c).

## Discussion

The nanoscopic SR X-CT observations showed that *fibre/plastic interface debonding* is initiated at positions with shorted interfibre distances (Fig. 3b,c). This behaviour occurs because a large interfacial normal stress is expected in this direction, as suggested by FEM calculations^26^. *Fibre/plastic interface debonding* occurs with different timings in the three directions of the indenter edges (orange lines, in Fig. 3a), which suggests that the interface bonding is not homogeneous among interfaces. Furthermore, it was also shown that *interface debonding* could accompany *in-plastic initiation* when the crack propagated in a specimen (*i.e*. 3D propagation) (Fig. 4d). A change in cracking modes could occur when the interface debonding strength is not homogenous or when cracks initiated at different locations meet each other. This finding of changes in cracking modes during propagation is only obtainable from 3D observations with a high spatial resolution such as those provided by nanoscopic SR X-CT.

In this study, it was found that *in-plastic crack initiation* was another cracking mode at an early stage of damage. The fracture behaviour, including *in situ* observation has studied extensively for the bulk plastic under mechanical load^37^, but few *in situ* observations have reported, because a higher spatial resolution is required. Cracking and pile-up formation have been reported for the thin film with SEM^38^. Figure 3c,d,f show that *in-plastic crack initiation* was induced by *interface debonding*, with the cracks then aggregating to form large cracks across the carbon fibres and plastic in the *X-Y* plane, which is almost parallel to the applied stress. In other words, both initiation mechanisms can occur or complete depending on the local situation such as local stress and the fibre array irregularities^26^, and also make initiated cracks propagate, resulting in formation of large cracks.

Furthermore, in-plastic crack propagation along the fibre directions (*Z*-direction) showed a variety of crack paths with smooth or rough surfaces (Fig. 4b,c). The fractured surfaces of bulk epoxy resin were investigated by linear elastic fracture mechanics and SEM and transmission electron microscopy (TEM), suggesting that smooth fracture zone was preceded and followed by crack initiation and crack arrest zones and that a step-like fracture was typified by the flow of internodular matrix during step^39^. The *in situ* observed in-plastic cracking interfaces were basically similar to the one observed in bulk, though the morphologies (smooth or rough surfaces) largely depend on the location. Thus, the difference in interface morphology can be attributed to the difference in local stress, deformation modes and strain rates, which is largely affected by the fibre array irregularities^26^. When the plastic thickness between carbon fibres was small (Fig. 4b), rough crack surfaces was observed. On the other hand, a smooth surface was formed in a thick plastic region (Fig. 4c). Different surface morphologies suggest that deformation modes and strain rates are not homogeneous but affected by many factors such as local stress and the fibre array irregularities^26^.

The two mechanisms of crack initiation: *debonding at fibre/plastic interfaces* and *cracking inside the plastic*, were revealed. Next question is whether they propagate equally in 3D within CFRP or not. This is difficult to answer in case of 2D observation of surfaces, where only one section or timing of damage was observed. The easiness of propagation in 3D can be evaluated by considering the location-dependent crack propagation susceptibility. Figure 5 shows crack width dependent on the *Z*-value (i.e. strain) at different locations. When a mechanical load was applied by the indenter, cracks formed near the indentation edge (*Z = *20 μm) and propagated along the *Z*-axis to a smaller *Z*-value. The *Z*-value corresponds to the strain, which was ~13% at *Z = *20 μm and ~0% at 0 μm. Thus, the slope, showing how easily crack width increases when strain was increases, corresponded to crack susceptibility; it was largely location dependent and showed which mechanism was dominant at each location.

Crack opening (or propagation) occurred with a gentler slope (low susceptibility) at locations with large distances between neighbouring fibres (lines L1, L2, and L3 in Fig. 5). In other words, ‘thick-plastic’ regions, where the deformation of the plastic resin could relax the increase in local stress around the crack tip, had low susceptibility to crack propagation. These regions featured propagation via *in-plastic crack initiation* in the *Z*-direction (Fig. 5d). These findings indicate possible measures that could be used to reduce crack initiation, such as regular alignment of carbon fibre with optimised ‘plastic thickness’, toughening the plastic with fine particles, or inserting particle-toughened interlayers between the plies^40^ to reduce in-plastic cracking.

By contrast, crack opening propagated with a steeper slope at locations where the distance between neighbouring fibres was small (lines L4 and L5 in Fig. 5). Therefore, ‘thin-plastic’ regions, where plastic deformation could not easily relax the increase in local stress around the crack tip, were highly susceptible to crack propagation. These regions displayed *fibre/plastic interface debonding* in the *Z*-direction (Fig. 5e).

In summary, we observed early-stage crack initiation and propagation in a CFRP *in situ* at a high spatial resolution (~50 nm) using nanoscopic SR X-CT with phase-contrast imaging. Our proposed technique revealed that voids and cracks do not simply result from local stresses but instead occur largely through two competing nanoscale mechanisms, namely, *fibre/plastic interface debonding* and *in-plastic crack initiation*, when the unidirectional ply specimen was mechanically loaded using a pyramidal indenter. The proposed method can be applied to observe crack initiation in CFRPs with different layups by changing the mechanical loading method from indentation to tensile, shear or bending stresses. Sample preparation is a considerable challenge for such investigation, but our current attempts have provided some preliminary results for tensile and shear stress tests.

Even under similar applied local stresses, the modes of crack initiation and propagation are strongly location dependent owing to parameters such as the plastic thickness between neighbouring fibres and the bonding strength at the fibre/plastic interface. These heterogeneities have been suggested to be related to many factors such as fibre array irregularities and the surface morphology and size of carbon fibres. Thus, the presented method provides an approach for improving our understanding of crack/void initiation and propagation mechanisms at multiple scales. In turn, such information is expected to facilitate the fabrication of CFRPs with reasonable safety margins for use in airplanes. The developed method can also be applied to investigate interfaces of adhesive bonded materials, which are of great importance in emerging fields such as adhesive manufacturing. Such investigations would provide helpful insights into the bonding of interfaces, which is key for controlling macroscopic properties.

## Methods

### Materials

The specimens consisted of T800H carbon/epoxy prepreg (fibre areal weight: 190 gsm, resin content: 35 wt%) with a layup of [90°/0°/90°/0°/90°]. The material was laid up and autoclave-cured using a standard airplane cure cycle (~450 K, a few hours). For the macroscopic X-CT measurements, the laminated plates were mechanically cut to produce 1 mm wide double-edged curved (bone-shaped) specimens with an overall gauge length of 1 mm (Supplementary Methods 1).

For the nanoscopic SR X-CT measurements, small specimens were prepared, because the field of view (FOV) in the case of SR X-CT was as small as 20 μm. Columnar specimens (1 mm long with a diameter of 60 μm) were prepared by mechanically cutting a single 0° ply CFRP specimen by moving a razor blade along the fibre direction so that the fibres were aligned in the Z-direction (Supplementary Methods 2). Machining damage was observed at a few locations within a few micrometres of the surface, but it caused no effects or artefacts in the observed images, because the specimen diameter (60 μm) is much larger than that of the FOV (20 μm).

### Macroscopic observations of cracks in the CFRP

Considering the hierarchical structure of CFRPs, we first examined crack formation at the millimetre scale *in situ* using macroscopic X-CT (Supplementary Methods 1). A bone-shaped specimen with a gauge of 1 mm^2^ was mechanically loaded under a tensile stress, and X-CT measurements were carried out under stress by maintaining the strain at a certain value. While monitoring the strain–stress curve, cycles of increasing the strain and X-CT measurements were carried out until the specimen fractured.

### Nanoscopic observations of crack initiation and propagation

At the nanoscale, crack/void initiation and propagation in CFRPs were monitored *in situ* using nanoscopic SR X-CT, newly installed at the NW2A beamline^41^ of the Photon Factory Advanced Ring (PF-AR) synchrotron facility (IMSS, KEK, Japan) (Supplementary Methods 2), which provides extremely intense, coherent single-photon-energy X-ray beams that are well suited to the nanoscopic SR X-CT with phase-contrast imaging (Supplementary Methods 3)^33–35^. In order to observe *in-plastic initiation* and/or *interface debonding*, SR X-CT must be combined with *in situ* observation techniques involving the sample preparation, nanomechanical testing, and measurement protocols that have been developed in this study.

*In situ* nanoscopic measurements were performed using a nanomechanical test stage^36^ to assess the initiation of transverse cracks in a 90° ply, which macroscopic X-CT observations revealed to be the fracture trigger site. The stress was applied to the specimen by moving it up to a pyramidal diamond indenter as a first-order experimental approximation of tensile loading (Fig. 1c, Supplementary Methods 2).

X-CT measurements were performed by rotating the test stage. Note that the obtained images represented the intrinsic features of crack initiation and propagation inside bulk CFRP specimens and were free of surface or thin-film effects because the FOV (20 μm in diameter, 40 × 2 μm in length) was much smaller than the specimen (60 μm in diameter, 1 mm in length). This is in contrast to previous electron microscopy imaging studies of CFRP surfaces or thin films, which displayed a different stress field from the bulk.

## Supplementary information

Supplementary Information

Supplementary Information2

## Data Availability

The authors will make available, upon request, typical data described in this work. It is understood that the data provided will not be for commercial use.
