# Supplementary material for: Nanoscopic origin of cracks in carbon fibre-reinforced plastic composites
Source: Sci Rep. 2019 Dec 17;9:19300. doi: 10.1038/s41598-019-55904-2 (PMC6917772; doi:10.1038/s41598-019-55904-2)
Supplement: Supplementary file 1 — Supplementary Information [file 41598_2019_55904_MOESM1_ESM.pdf]

Supplementary information

# Nanoscopic origin of cracks in carbon fibre–reinforced plastic composites

**Authors**

Masao Kimura*[1, 2], Toshiki Watanabe[1], Yasuo Takeichi[1, 2], Yasuhiro Niwa[1]

**Affiliations**

[1]Photon Factory, Institute of Materials Structure Science, High Energy Accelerator Research Organization (KEK), Tsukuba, Ibaraki 305-0801, Japan.

[2]Department of Materials Structure Science, School of High Energy Accelerator Science, SOKENDAI (Graduate University for Advanced Studies), Tsukuba, Ibaraki 305-0801, Japan.

*Corresponding author. E-mail: masao.kimura@kek.jp

**Table of Contents**

**Supplementary Methods**
- **1 *In situ* observations with macroscopic X-CT**
- **2 *In situ* observations with nanoscopic SR X-CT**
- **3 Phase-contrast imaging**

**Supplementary Notes**
- **1 *In situ* macroscopic X-CT measurements**
- **2 *In situ* nanoscopic SR X-CT measurements**

**Supplementary Methods**

Considering the hierarchical structure of CFRPs, we first examined cracking in a large specimen by *in situ* observations using macroscopic X-CT and identified the first step in the crack-initiation mechanism. Then, we focused on crack initiation in a small specimen by *in situ* observations using nanoscopic X-CT using synchrotron-radiation (nanoscopic SR X-CT) because the field of view (FOV) in the case of SR X-CT was as small as 20 µm (Supplementary Table S1).

**Supplementary Methods 1: *In situ* observations with macroscopic X-CT**

Preliminary macroscopic X-CT measurements were carried out using an in-house X-ray source (Zeiss Xradia 520 Versa, Carl Zeiss Microscopy GmbH) and a mechanical test stage (Deben CT5000-TEC, Carl Zeiss Microscopy GmbH). These measurements were performed *in situ* for specimens subjected to mechanical stress (Supplementary Fig. S1). Typical measurement conditions are shown in Supplementary Table S1. The results provided FOVs to be measured for nanoscopic SR X-CT observations.

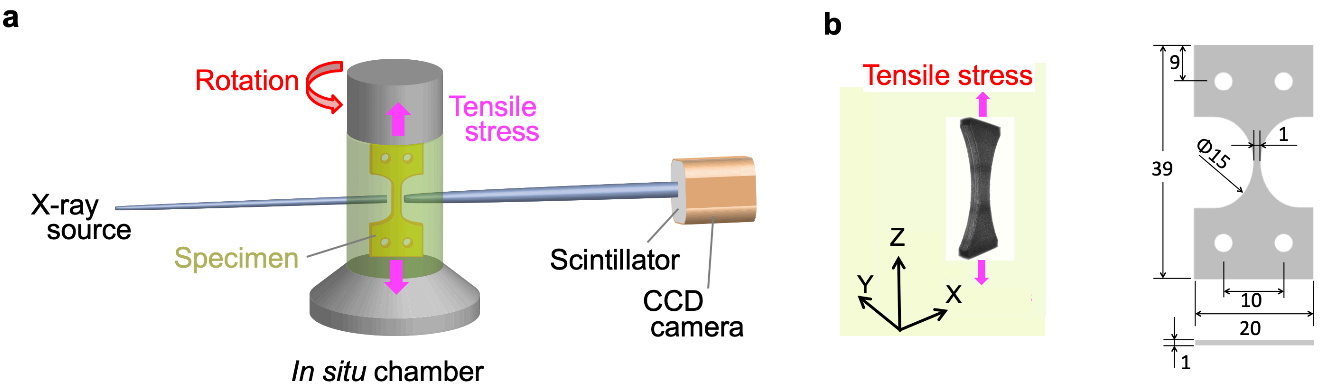


**Supplementary Fig. S1. *In situ* observations with macroscopic X-CT. a**, Schematic representation of the cell used for *in situ* mechanical testing. **b**, Typical specimen dimensions in millimetres.

**Supplementary Table S1.** Typical conditions for X-CT and SR X-CT measurements

| | Macroscopic X-CT | Nanoscopic SR X-CT |
|---|---|---|
| X-ray energy (keV) | 80–50 (peak), 27–17 (mean) | 8 (monochromatic) |
| Voxel size [and FOV] | 0.7 µm [5 mm]–20 µm [100 mm] | 39 nm [20 µm] or 78 nm [40 µm] |
| Number of radiographs | 720–5000 | 150–600 |
| Range of rotation angle (°) | 0–360 | 0–150 (180) |
| Exposure time (s) | 1–10 | 1–10 |
| Total scan time (min) | 60–600 | 10–120 |

**Supplementary Methods 2: *In situ* observations with nanoscopic SR X-CT**

Supplementary Figure S2 shows the experimental setup for nanoscopic SR X-CT measurements. A monochromatic X-ray beam was focused onto the sample using an elliptical glass capillary, and the image was projected onto the scintillator using a Fresnel zone plate (ZP) lens that provided a magnification of ~70× at 8 keV. The obtained image was further magnified by an optical lens (~20×) and recorded using a charge-coupled device camera. The total spatial resolution, as determined by the Siemens star pattern, was ~50 nm. Phase-contrast imaging (Supplementary Fig. S3)[1], which relies on the scattering of X-rays at interfaces to enhance image contrast at fibre/plastic interfaces, was performed using a phase ring located between the ZP and the scintillator.

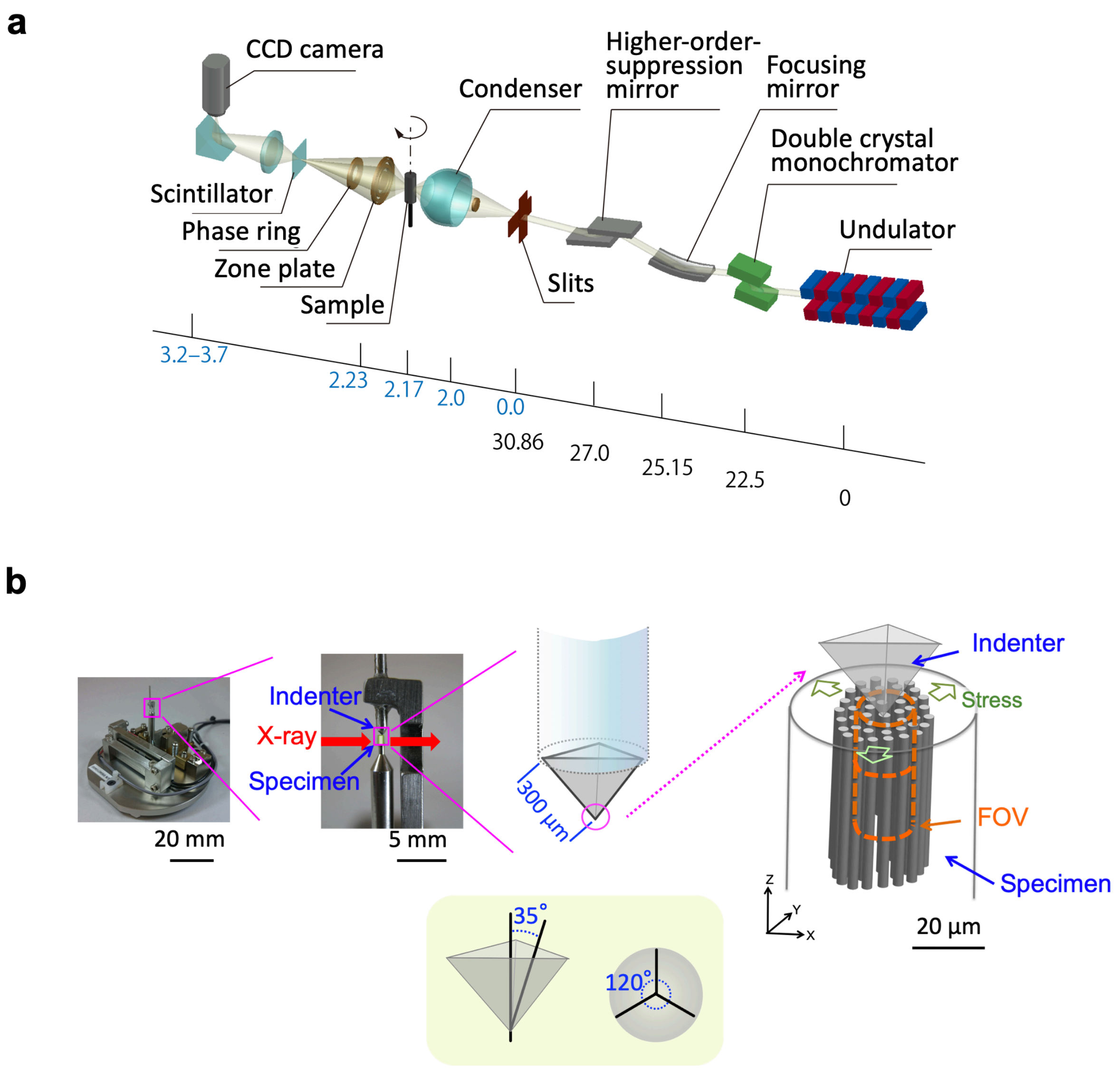


**Supplementary Fig. S2. Experimental setup. a**, Diagram of the SR X-CT instrument. Black and blue numbers are guides for scales (note that the scale is not linear). **b**, Optical images (left) and schematic illustration (right) of the nanomechanical test stage. The FOV in the nanoscopic SR X-CT measurements (orange broken lines) was much smaller than the specimen dimensions (grey lines), which eliminated surface effects in the acquired image.

The typical experimental conditions for *in situ* nanoscopic SR X-CT[*)] measurements are provided in Table S1. The temperature around the specimen was controlled within 0.1 K for several hours. The rotation error was corrected using an offset table obtained by preliminary measurements employing a reference needle, and the acquired images were then aligned using the Au reference balls (a few micrometres in diameter) to correct dilatation during rotation. Finally, volume data were reconstructed into 3D images using the filtered back-projection (FBP) method. Iterative reconstruction was also attempted, but there was no significant difference in the reconstructed images under typical measuring conditions. Therefore, FBP was mainly used for image reconstruction.

The high spatial resolution (~50 nm in 3D) of the system was confirmed using a standard test pattern (Siemens pattern). To obtain a wide FOV in the *Z*-direction, along which a crack propagates, consecutive X-CT measurements were carried out, and the two volume data sets were merged into one (20 μm in diameter, 20 × 2 μm in length). Images at the interfaces between fibres, resin, and dispersed particles were enhanced by phase-contrast imaging, which relies on the scattering of X-rays at interfaces[1,2] (Supplementary Fig. S3). Therefore, images of the carbon fibres, matrix plastics, and voids/cracks showed clearly enhanced boundaries at a spatial resolution of ~50 nm.

Supplementary Fig. S2b shows the nanomechanical test stage[3] designed specifically for the nanoscopic SR X-CT measurements. The stage featured a high-precision piezo actuator and an integrated load cell. The specimen was mounted on the bottom anvil with an adhesive material. Combinations of different types of tip geometries and the up and down movements of the bottom anvil can provide various mechanical testing modes, such as compressive, tensile, and bending tests. In this study, the pyramidal diamond indenter was located at the top anvil. The details of the indenter are shown in Supplementary Fig. S2; the diamond tip was mounted on a steel rod with a diameter of 1 mm, and the tip was mechanically polished into a predetermined shape. Finally, the surface was smoothly polished to minimize the friction between the indenter and a specimen. After polishing, angles of 120.00°, 120.00°, 120.00°, 35.11°, 35.09°, and 35.12° were measured for the indenter. The applied stress was not simple tensile or Mode I stress, but may have included Mode II stress and the frictional interactions between the tip and the sample. However, we used the pyramidal diamond indenter as a first trial because of the relative experimental simplicity. This nanomechanical test does not replicate a tensile test exactly, but we assume that the observed results contain phenomena that would be found in experiments under pure Mode I stress, the investigation of which is now an on-going project.

The applied force, caused by moving the sample up against the indenter, was monitored using a force gauge. The maximum load of 9 N corresponded to a stress of ~9 GPa under the experimental conditions, which enabled efficient recording of the displacement–load curve and its association with the microstructure evolution observed in the corresponding 3D tomographic reconstructions. Notably, the X-CT FOV (20 μm in diameter) was much smaller than the specimen (60 μm in diameter), leading to a FOV image devoid of surface effects. This is a major

---

[*)] Note: SR X-CT is also called X-ray absorption fine structure CT (XAFS-CT) because 3D X-CT images for each voxel can be obtained from XAFS spectra recorded within an energy range of 5–11 keV.

advantage of nanoscopic SR X-CT over electron microscopy, in which surface or thin film effects, such as stress release, are inevitable.

**Supplementary Methods 3: Phase-contrast imaging**

When an object is illuminated by a parallel X-ray beam, the detector captures a transparent image by absorption contrast (Supplementary Fig. S3a)[1, 2]. Simultaneously, beams diffracted at a slight angle at interfaces such as carbon fibre/plastic, fibre/crack, and plastic/crack interfaces form positive and negative fringes at the boundary, which can be observed by absorption contrast. In nanoscopic SR X-CT measurements, the effects of phase contrast can be enhanced using a phase ring made of gold with a thickness of 1.33 μm (Supplementary Fig. S3b), which shifts the phase of the direct beam by $\lambda/4$. Transparent and diffracted images are enhanced relative to each other (Supplementary Fig. S3c). As clearly shown in Supplementary Fig. S3d, phase-contrast imaging enhances contrast (white/black fringes) at the carbon/air and Au/air interfaces.

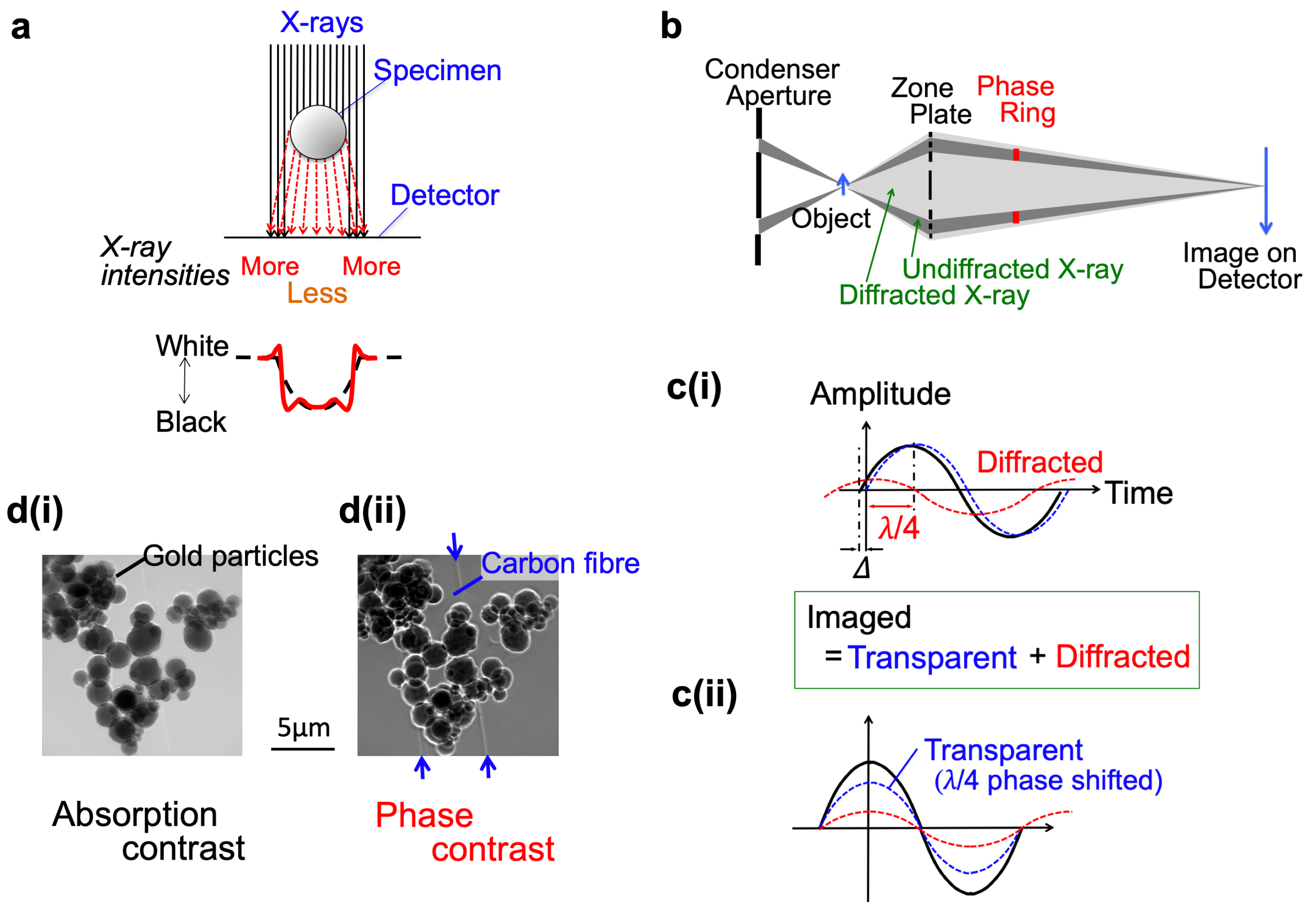


**Supplementary Fig. S3. Phase-contrast imaging. a**, Schematic representation of absorption and phase-contrast imaging using direct (black arrows) and diffracted beams (red arrows). **b**, X-ray optics for phase-contrast imaging. **c(i)**,Time evolution of the transmitted (blue), diffracted (red), and transmitted + diffracted radiation intensities (black) without a phase ring. **c(ii)**, Model case where a phase ring is used. **d**, Transparent images of Au particles on a carbon fibre acquired by (i) absorption imaging (without a phase ring) and (ii) phase-contrast imaging (with a phase ring). Interfaces between the carbon fibres and air are clearly observed only when the phase ring was used, as shown by blue arrows in **d(ii)**.

**Supplementary Notes**

**Supplementary Note 1: *In situ* macroscopic X-CT measurements**

First, strain–stress curves were measured without X-CT (red solid line, Supplementary Fig. S4a) by continuously increasing the strain at a rate of 0.1 mm/min until fracture to determine the fracture strength ($\sigma_f$). The strain was measured and corrected using a strain gauge attached to the specimen. The strain–stress curve was linear and showed a yield before fracture, which are typical features of this system.

Subsequently, *in situ* X-CT measurements were performed at stresses of 0.36, 0.61, and 0.92$\sigma_f$ (blue broken line, Supplementary Fig. S4a). The results showed that transverse cracks of a few hundred micrometres first appeared in the 90° plies at ~40–50% of the fracture strength ($\sigma_f$), and their propagation accelerated several times with an increase in the tensile stress (S-2, Supplementary Fig. S4b). At higher stresses, these transverse cracks induced cracking in neighbouring 0° plies (fibre splitting) and grew along the fibres to form macroscopic cracks (blue ovals in S-3, Supplementary Fig. S4b). Finally, these microscopic cracks propagated along the fibres and grew into macroscopic cracks larger than millimetres (S-4, Supplementary Fig. S4b). Macroscopic *in situ* X-CT observations showed that transverse cracks were initiated in the 90° plies, which is the FOV to be measured on the nanoscale using SR X-CT.

Drops in stress were observed when the strain was maintained at each value. No image blurring was observed, showing that there were no large displacements within the specimen that exceeded the spatial resolution (~10 μm). Thus, the stress drops may be caused by changes, such as polymer relaxation, at the crack tip at high strains, which occur on a scale smaller than the spatial resolution.

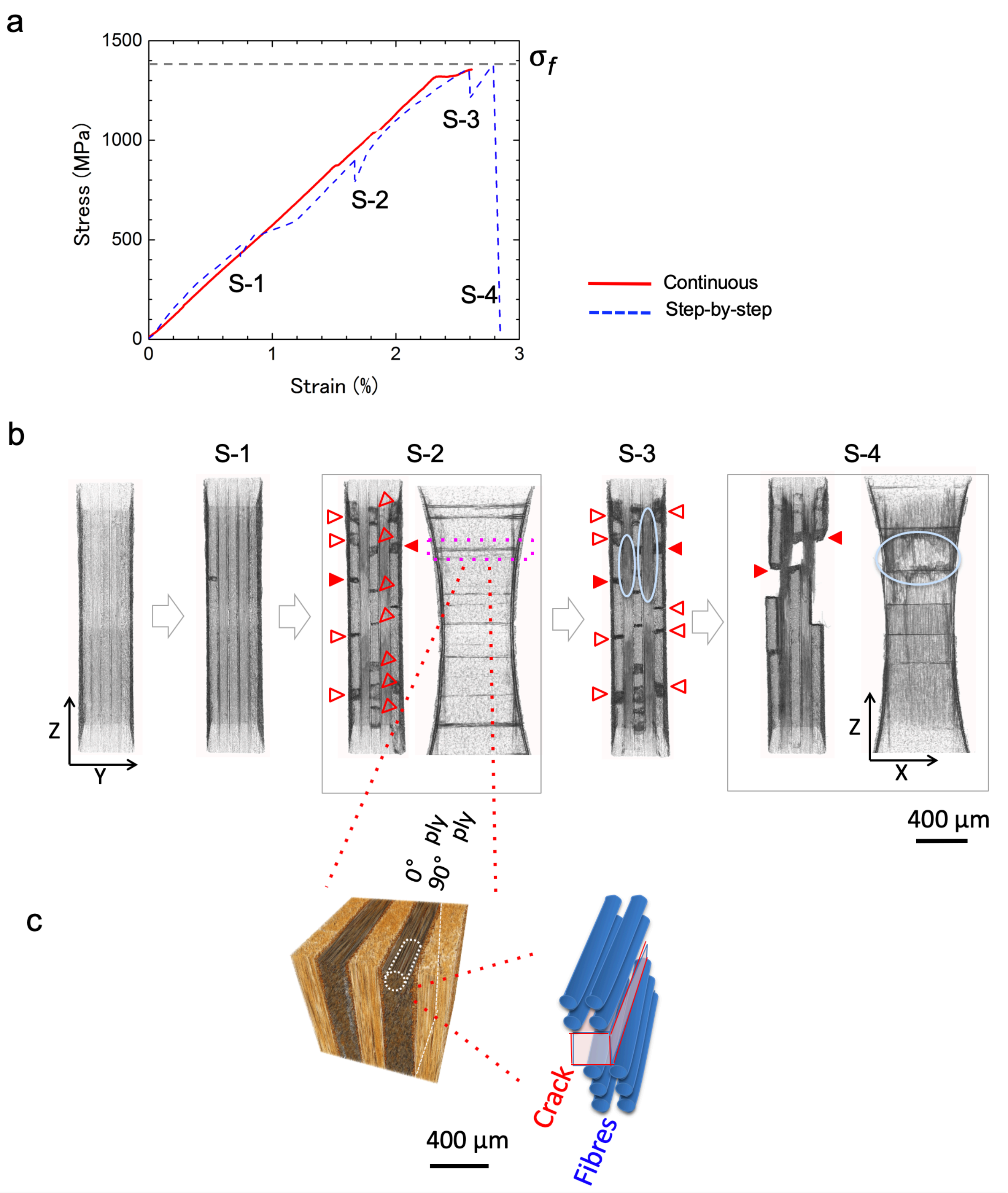


**Supplementary Fig. S4. Macroscopic crack observation by X-CT. a**, Strain–stress curves of the CFRP specimen ([90°/0°/90°/0°/90°]). **b**, Cross-sectional images reconstructed from X-CT data at strains of 1.1% (S-1), 2.2% (S-2), and 3.5% (S-3). Initially, transverse cracks appeared in the 90° plies (red triangles in S-2 and S-3), where stress was applied in the weakest prepreg direction (perpendicular to the carbon fibres). The transverse cracks indicated by closed triangles are those that eventually triggered macroscopic fracture. The blue ovals in S-3 show splitting in the central 0° plies. **c**, Schematic of a transverse crack in the 90° plies, which is the FOV to be measured with nanoscopic SR X-CT. A cylindrical CFRP specimen (broken lines) was mechanically cut out for the nanoscopic SR X-CT measurements.

**Supplementary Note 2: *In situ* nanoscopic SR X-CT measurements**

Two dimensional (2D) time-lapse observations were performed to confirm that crack propagation occurred using the nanomechanical test stage. 2D transparent images were monitored at intervals of 0.5–1.0 s under stress. Slight but significant changes were observed when the insertion distance was increased. These changes corresponded to crack propagation, although it was difficult to identify the shape of the crack clearly. Supplementary Fig. S5 shows a typical example of the changes observed in the 2D transparent images after the load reached $\sigma_{ini.}$ at $t = 0$ ('1' in Fig. 2). Slight but significant changes were observed at $t = 0$–9 s, showing the stepwise propagation of the crack with a step of approximately 1 μm. These results show that we measured the initiation and propagation of cracks by pushing the pyramidal indenter into a specimen instead of simply measuring friction between the indenter tip and a macroscopic crack already exited in the specimen. This was also confirmed by observation of 3D images obtained with nanoscopic SR X-CT (Figs. 3 and 4, Supplementary Movie S1).

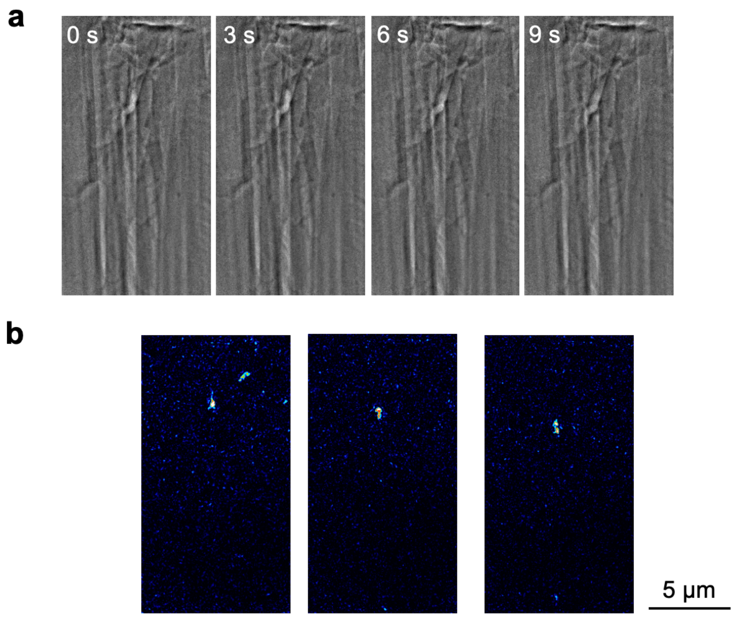


**Supplementary Fig. S5. Time-lapse transmission images obtained by *in situ* nanoscopic SR X-CT. a**, Images measured at $t$ = (i) 0, (ii) 3, (iii) 6, and (iv) 9 s, where $t = 0$ corresponds to the time when the load reached $\sigma_{ini.}$ ('1' in Fig. 2). **b**, Differences between successive images shown in **a**.

**Supplementary Movie S1. Crack propagation in CFRP.**

Segmented 3D images of cracks formed in the CFRP in a stable state. Purple and yellow pixels represent carbon fibres/polymer resin and cracks (air), respectively. Segmentation was carried out for the reconstructed nanoscopic SR X-CT-derived volume data when cracks reached a stable state under the applied stress ('1' in Fig. 2). It is clearly shown that cracks propagate in a complicated 3D manner.

## References

1. Buffière J.-Y., Maire, E., Cloetens, P., Lormand, G. & Fougères, R. Characterization of internal damage in a $MMC_p$ using X-ray synchrotron phase contrast microtomography. *Acta Mater.* **47,** 1613–1625 (1999).

2. Cloetens, P. et al. Observation of microstructure and damage in materials by phase sensitive radiography and tomography. *J. Appl. Phys.* **81,** 5878–5886 (1997).

3. Patterson, B. M. et al. *In situ* laboratory-based transmission X-ray microscopy and tomography of material eeformation at the nanoscale. *Exp. Mech.* **56,** 1585–1597 (2016).
